# Between equilibrium and chaos, with little restitution: a narrative analysis of qualitative interviews with clinicians and parent carers of children with medical complexity

**DOI:** 10.1186/s12913-024-10973-6

**Published:** 2024-04-23

**Authors:** Stephanie Hodgson, Kirsten Noack, Ashleigh Griffiths, Michael Hodgins

**Affiliations:** 1 Hunter New England Local Health District, Newcastle, Australia; 2 School of Women’s and Children’s Health, University of New South Wales, Sydney, Australia

**Keywords:** Children with medical complexity, Narrative analysis, Care coordination, Paediatrics

## Abstract

**Background:**

Children with medical complexity (CMC) comprise 1% of the paediatric population, but account for over 30% of health service costs. Lack of healthcare integration and coordination for CMC is well-documented. To address this, a deep understanding of local contextual factors, experiences, and family-identified needs is crucial. The aim of this research was to investigate the lived experiences of CMC, their families, and healthcare staff, focusing on understanding the dynamics of care coordination and the challenges faced in providing integrated care, in order to inform the development of effective, family-centred models of care.

**Methods:**

In April to July 2022, 31 semi-structured interviews were conducted with parents/guardians of CMC and healthcare professionals who care for CMC. Interviews explored complex paediatric care and care coordination barriers. An inductive thematic analysis was undertaken. Themes were then further explored using Frank’s narrative approach.

**Results:**

Through analysis, we identified that the restitution typology was absent from both staff and parent/guardian narratives. However, we uncovered narratives reflective of the chaos and quest typologies, depicting overwhelming challenges in managing complex medical needs, and proactive efforts to overcome barriers. Importantly, a novel typology termed ‘equilibrium’ was uncovered. Narratives aligning with this typology described medical complexity as a balance of power and a negotiation of roles. Within the equilibrium typology, illness trajectory was described as a series of negotiations or balancing acts between healthcare stakeholders, before finally reaching equilibrium. Participants described seeking a balance, where their expertise is respected, whilst maintaining the ability to rely on professional guidance and support. These insights provide a nuanced understanding of the multifaceted narratives shaping care experiences for CMC and their families.

**Conclusions:**

Our research delineates multifaceted challenges within the care landscape for CMC, their families, and healthcare staff. Embracing the equilibrium narrative typology highlights the criticality of tailored, integrated care models. This necessitates prioritising clear role delineation and communication among caregivers, implementing support systems addressing the challenges of continuous caregiving, and integrating parents/guardians as essential members of the care team. These insights advocate for pragmatic and sustainable strategies to address the unique needs of CMC and their families within healthcare systems.

**Supplementary Information:**

The online version contains supplementary material available at 10.1186/s12913-024-10973-6.

## Background

Children with medical complexity (CMC) account for 1% of the total paediatric population yet contribute to over 30% of health service costs [1]. CMC require frequent access to a variety of health services and team-based interdisciplinary care over many years [2]. CMC are characterised, in part, by their ongoing reliance on multiple services. CMC, who may also be known as “complex chronic” [3] have multiple significant chronic health problems that affect multiple organ systems, resulting in functional limitations, high health care need or utilisation, and often the need for medical technology [4–6]. 

In most high-income countries, the team around the child is vast and varied and can include doctors, nurses, allied health workers, disability support workers, social services and many more. Crucial to this team around the child is the parent/guardian. The disconnect between these stakeholders and the overall lack of integration of healthcare for CMC is well documented [7–10]. The idea of designing integrated services to meet the needs of this growing population is also well documented [11]. We anticipate the precursor to designing new models of care for this cohort is a deep understanding of what is common and what is consistent between and amongst staff and families. But also, what is common and consistent amongst this medically complex cohort, where the diagnoses, touchpoints and experiences are varied.

To create a robust and appropriate co-designed model of care, a deep understanding of the local contextual factors, beliefs and experiences of key parties is crucial. Routine quantitative data sets cannot fully capture the experience of treating or caring for CMC [4]. New models dedicated to CMC must consider local contextual factors, expertise, existing care access, and community resources [4]. Subsequent novel models will be inadequate in scope if they cannot incorporate family-identified needs and functional limitations [4]. 

This qualitative research aims to understand the experiences of CMC and their caregivers through illness narratives. Semi-structured interviews provide a means of unlocking rich information about the providers and receivers of healthcare [12]. Drawing upon previous research in the field, this study explores key themes that emerge from the narratives of both staff and parents involved in the care of CMC. These narratives offer a valuable perspective, capturing the intertwined experiences of various stakeholders, with closely aligned yet distinct perspectives on the care provided to CMC. By delving into the rich stories shared by staff and parents, we can gain deeper insights into the challenges faced by this cohort and identify potential areas for improvement within the healthcare system.

## Methods

### Setting

The study took place in Hunter New England Local Health District (HNELHD) in New South Wales, Australia. HNELHD serves a diverse population of over 920,000 individuals, including a significant proportion of Aboriginal and Torres Strait Islander people and residents born overseas. HNELHD includes a major metropolitan centre as well as several large regional centres, smaller rural centres, and remote communities [13]. The paediatric specialty hospital in the district is the John Hunter Children’s Hospital (JHCH), located in Newcastle. The JHCH provides specialised tertiary referral services for complex paediatric care, including medical, surgical, trauma, and neonatal services and admit over 10,000 children and young people to hospital each year and perform over 100,000 occasions of service through outpatient clinics [14]. 

### Recruitment

Staff involved in the care of CMC and parents or guardians of CMC were recruited during the period April to July 2022. Printed advertising materials about the project were displayed in HNELHD health facilities, schools and early childhood education and care centres. The project was also promoted on local community organisation and hospital social media channels. Additionally, health staff were directly informed about the study and provided with both printed and digital materials to share with colleagues and patients. Interested individuals were instructed to register their interest in participating, enabling self-referral for potential health staff, parent/guardian, and child/young person participants. A purposive sample was then selected from this list. Eligible health staff participants worked within HNELHD and were involved in the care of CMC. Likewise, parent/guardian participants were eligible if they were the primary caregivers of CMC receiving care in HNELHD. Child/young person participants, aged 7–17 years, with medical complexity and receiving care in HNELHD, were also eligible.

To select participants, the project team conducted brief screening phone calls using predefined questions outlined in Table 1. These questions, tailored for staff, gathered details regarding their engagement with CMC, workplace, responsibilities, and experience. Similarly, questions for parents/guardians and children/young people aimed to ensure a diverse representation from both rural and metropolitan areas, covering various medical conditions. For eligibility purposes, age was asked during screening for child/young person participants only.

**Table 1:** Participant screening questions

Participant group	Screening questions
Healthcare staff participant	1. Do you work with medically complex children? 2. Where do you work? 3. What is your role? 4. How many years experience do you have in this role and similar roles?
Parent/guardian and child/young person participant	1. What is your/your child’s diagnosis? 2. What specialty teams/care do you/your child access? 3. Where do you/your child live? 4. What is your/your child’s age*

Screening questions asked of potential healthcare staff, parent/guardian and child/young person participants to ensure a purposive sample for interview

* Only the age of the child was requested

Following screening, ten parents/guardians of CMC, one CMC and 21 health professionals who work with or manage CMC were selected to participate. 60% of interviewed participants resided or worked within metropolitan settings and 40% in rural/regional settings. All sectors of the district and major facilities were represented. We did not record details on gender or years of experience.

One participant withdrew from the study prior to being interviewed due to illness. Unfortunately, this was the only CMC participant and no further children/young people were recruited. This was due to the young age of the child, parent preference for participating without their child present and healthcare issues that limited the ability of the child to participate in an interview.

Further detail of participant characteristics can be found in Tables 2 and 3.

Participants received information sheets outlining the project’s objectives, participation requirements, associated risks, and benefits. Tailored versions were prepared for healthcare professionals, parent/guardians, and children aged 7–13 and 14–17. Written consent was obtained from all participants before conducting interviews.

### Data collection

Thirty-one interviews were conducted between April and July 2022. Interviews were conducted with staff and parents/guardians from across HNELHD. One Health Service Project Officer and one Health Service Project Manager conducted the interviews. The interviewers did not have any pre-existing relationship with the participants. The duration of the interviews ranged from 45 to 85 min.

**Table 2:** Parent/guardian and child/young person participant characteristics

Participant	Role within family unit	Residential location (RRMA)*	Tertiary specialties involved in their child’s care
P1	Mother	Metropolitan	Neurology, Gastrology, General paediatrics
P2	Mother	Metropolitan	Respiratory, Endocrinology, Gastrology
P3	Mother	Metropolitan	Neurology, Gastrology, General paediatrics
P4	Mother	Metropolitan	General paediatrics, Respiratory, Endocrinology, Gastrology
P5	Mother	Metropolitan	Neurology, Oncology, General paediatrics
P6	Mother	Metropolitan	Palliative care, Neurology, Respiratory, General paediatrics
P7	Mother	Metropolitan	Endocrinology, Ophthalmology, Orthopaedics, General paediatrics
P8	Mother	Rural	Neurology, Gastrology, General paediatrics
P9	Mother	Rural	Orthopaedics, Rehabilitation, Urology, General paediatrics
P10	Father	Rural	Neurology, Orthopaedics, General paediatrics

Characteristics of the 10 parent/guardian participants, including their role within the family unit, the residential location of their child, and the tertiary specialty teams involved in their child’s care

* The Rural, Remote and Metropolitan Area (RRMA) classification divides Australia metropolitan, rural and remote zones [15]

**Table 3:** Health professional characteristics

Participant	Professional role	Workplace location (RRMA)*
S1	Clinical Nurse Educator	Rural
S2	Clinical Nurse Consultant	Metropolitan
S3	Medical Consultant	Metropolitan
S4	Medical Consultant	Metropolitan
S5	Service Manager	Rural
S6	Service Manager	Metropolitan
S7	Clinical Nurse Consultant	Metropolitan
S8	Clinical Nurse Consultant	Metropolitan
S9	General Practitioner	Rural
S10	Other Senior Nurse	Rural
S11	Occupational Therapist	Metropolitan
S12	Physiotherapist	Metropolitan
S13	Child Life therapist	Metropolitan
S14	Clinical Nurse Consultant	Rural
S15	Clinical Nurse Consultant	Rural
S16	Clinical Nurse Consultant	Rural
S17	Medical Consultant	Rural
S18	Physiotherapist	Rural
S19	Dietician	Rural
S20	Service Manager	Metropolitan
S21	Social Worker	Rural

Characteristics of the 21 health staff participants, including their professional role and workplace location

* The Rural, Remote and Metropolitan Area (RRMA) classification divides Australia metropolitan, rural and remote zones [15]

Interviews were deemed the most suitable approach for data collection, over other approaches including focus groups, due to both the logistical difficulty and the likelihood that participants may choose to withhold information in the presence of other staff [16]. The study was approved by the Hunter New England Human Research Ethics Committee (2022/ETH00104). Semi-structured interviews were conducted with staff and parents/guardians to collect qualitative data on the lived experience of complex paediatric care and the perceived barriers and enablers for consistent care coordination. The semi-structured interview guides, developed by the project team [see Additional file 1], were piloted with two nursing staff and one project consumer representative who was the mother of a CMC. Questions were centred on staff and parent/guardian experience in caring for CMC, including typical interactions, home or work life and perceived barriers and enablers to optimal care. As the interview questions required participants to recall potentially difficult experiences, interviewers offered breaks, provided the option to stop at any time and connected participants with information on mental health support services as appropriate.

One-on-one interviews were conducted virtually to prioritise participant convenience and comfort, with flexible scheduling allowing for sessions to be held at times and locations most convenient for participants. Each participant was interviewed once. Data collection for the qualitative interviews continued until data saturation, as agreed by the study team based on the likelihood of gaining additional, pertinent information from subsequent interviews. Interviews were recorded, transcribed verbatim and de-identified. Transcripts were not returned to participants for comment or correction. Pseudonyms and broad descriptors of people participating (for example, “physiotherapist”, “nurse”, “mother”) were assigned to maintain context and perspective of the participant whilst protecting privacy.

### Analysis

Two project members carried out an inductive thematic analysis following Braun and Clarke’s approach [17]. The analysis involved independently generating codes from the first transcript, engaging in discussion around discrepancies and reaching a consensus on a draft coding hierarchy. The project team collectively refined and developed the hierarchy during the coding of the remaining 30 transcripts. Once all transcripts were coded, further discussions between authors led to the generation of a thematic structure which was further refined during repeated engagement with the data, to ensure that the themes generated resonated with the story of the complete data set. NVivo 11 was used to assist with the organisational aspects of the analysis.

Given the alignment of our findings with existing narrative approaches [18], our initial themes were then further explored using Frank’s narrative approach as a second phase of analysis [18]. Storytelling and narrative analysis have long been popular within the social sciences. ‘Narratives’ can be understood in many ways, but traditionally they have been understood as a retold chronology of events with a plot and set of characters. Narratives often evoke causality, explaining a complex event or series of events coherently in terms of an underlying theory or ontology that the teller imparts [19]. Narratives engender coherence when explaining complex sets of events by ascribing meaning and enabling temporal connection to the complexity, ambiguity, and unpredictability of social and organisational experiences [20]. The importance of narratives is reflected in healthcare, where clinicians consistently entwine clinical information into stories of personal experience to share knowledge and generate effects [21]. A narrative approach offers a different way of exploring a patient’s world and reflecting on our everyday practices [22]. Storytelling is a particularly important tool for “reporting and illuminating the cultural contexts of health: the practices and behaviour that groups of people share and that are defined by customs, language and geography” [23]. 

Frank’s framework includes the restitution narrative, a narrative typology categorising participants’ stories where illness is described as a temporary disruption that can be overcome through effort and determination. Within the restitution typology, participants may see their illness as a challenge or a test, and they may be motivated to take action and make changes in order to restore their health and well-being. The quest narrative categorises participant’s illness stories as a journey or a quest in which they may be motivated to search for answers, solutions, or cures. They may see their illness as a mystery that needs to be solved, and they may be willing to try new treatments or approaches to find relief. The chaos narrative categorises participants’ stories of illness as overwhelming and unpredictable, and they may feel helpless and out of control. They may see their illness as a source of chaos or disorder in their lives and may struggle to find meaning or purpose in their experiences. The project team considered the different experiences and themes identified within the data set through these typologies to understand how clinicians and families consider their perceptions and experiences of illness trajectories for CMC. Through analysis, the project team identified that the restitution typology was notably absent from the narratives of both staff and parents/guardians. In addition, the team identified one new narrative typology prevalent in the narratives of participants. The team named this new typology ‘equilibrium’. As such, findings are presented against the narrative typologies of chaos, quest, and equilibrium. To provide a more in-depth understanding, each typology will first be demonstrated with an exemplar story in the results section below.

## Results

### Chaos typology

*From the moment Lucas was diagnosed with complex respiratory issues at 6 months old, his mother Emma’s life took a chaotic turn. Formerly a corporate accountant, she made the difficult decision to leave her job and dedicate herself to caretaking responsibilities. Initially overwhelmed, Emma sought guidance from nurses who assured her that she would eventually become adept at managing her child’s condition. However, over time, she came to the realisation that the sense of control she had hoped for would never eventuate: “it’s a humbling realisation, to know that disorder will forever be a part of our lives”. Despite her best efforts to gather information and ask questions during appointments, Emma often left feeling unheard and ill-equipped to provide the necessary care. The constant juggling of appointments and caregiving responsibilities strained the family dynamics, leaving Lucas’s sister feeling overlooked and resentful of the attention her sibling received.*

The chaos narrative typology acknowledges the overwhelming nature of managing the complex medical needs of children (see Table 4 for themes identified within the chaos typology). Parents told stories of coming away from appointments feeling they had not received all the information they needed, despite having made notes and asking questions. Managing complex medical needs can feel like a full-time job in addition to other responsibilities, and parents felt overwhelmed by the sheer volume of information that they received. This often led to stories of disengagement and a lack of trust in medical professionals. This, in turn, diminished confidence in their ability to meaningfully contribute to the journey of care.

**Table 4:** Sub-themes and themes present within each narrative typology

Subtheme	Theme	Narrative typology
Feeling inundated by the demands of caring for a child with complex medical needs	Overwhelmed caregivers	Chaos
Experiencing emotional and physical strain due to caregiving responsibilities	Overwhelmed caregivers	Chaos
Managing the financial burden associated with medical expenses	Overwhelmed caregivers	Chaos
Coping with disruptions to daily routines and family dynamics	Overwhelmed caregivers	Chaos
Navigating a disjointed healthcare system with numerous providers and services	Fragmented healthcare system	Chaos
Dealing with administrative hurdles and delays in obtaining necessary services and supports	Fragmented healthcare system	Chaos
Experiencing gaps in continuity of care and communication between different healthcare providers	Fragmented healthcare system	Chaos
Struggling to access specialised care and resources due to geographical or financial barriers	Fragmented healthcare system	Chaos
Struggling to obtain accurate and timely information about the child’s condition and treatment options	Lack of information and communication	Chaos
Experiencing breakdowns in communication between healthcare providers and caregivers	Lack of information and communication	Chaos
Feeling overwhelmed by the volume of medical information and terminology	Lack of information and communication	Chaos
Coping with feelings of grief, guilt, and anxiety related to the child’s illness	Emotional toll on families	Chaos
Managing the emotional impact of witnessing the child’s suffering and medical procedures	Emotional toll on families	Chaos
Balancing the needs of the sick child with those of other family members	Emotional toll on families	Chaos
Navigating complex family dynamics and relationships under stress	Emotional toll on families	Chaos
Juggling multiple caregiving responsibilities, including medical, emotional, and logistical tasks	Role overload	Chaos
Struggling to balance caregiving duties with other responsibilities, such as work, household chores, and caring for other family members	Role overload	Chaos
Experiencing feelings of inadequacy and guilt for not being able to meet all of their child’s needs	Role overload	Chaos
Living with the uncertainty of their child’s prognosis and future health outcomes	Uncertainty and fear	Chaos
Coping with the fear of medical complications, hospitalisations, and unexpected emergencies	Uncertainty and fear	Chaos
Facing anxiety and worry about the long-term implications of their child’s condition on their family’s life	Uncertainty and fear	Chaos
Empowering caregivers to advocate for the child’s needs within the healthcare system	Advocacy and empowerment	Quest
Providing caregivers with the tools and resources to navigate complex healthcare processes	Advocacy and empowerment	Quest
Fostering partnerships between caregivers and healthcare providers to ensure the child’s voice is heard	Advocacy and empowerment	Quest
Fostering collaborative relationships between caregivers and healthcare professionals	Collaboration with Healthcare Providers	Quest
Engaging in open and transparent communication with healthcare providers	Collaboration with Healthcare Providers	Quest
Participating in shared decision-making processes regarding the child’s care	Collaboration with Healthcare Providers	Quest
Working together to develop comprehensive care plans tailored to the child’s unique needs	Collaboration with Healthcare Providers	Quest
Negotiating with healthcare providers to access necessary services and supports	Negotiation and persistence	Quest
Advocating for the child’s needs in challenging or resistant healthcare environments	Negotiation and persistence	Quest
Seeking alternative solutions and pathways when traditional approaches prove ineffective	Innovation and creativity	Quest
Harnessing technology to enhance communication and coordination of care	Innovation and creativity	Quest
Celebrating milestones and successes in the child’s care journey	Sense of achievement and fulfilment	Quest
Finding purpose and meaning in the caregiving role	Sense of achievement and fulfilment	Quest
Recognising and appreciating personal growth and development through caregiving experiences	Sense of achievement and fulfilment	Quest
Adapting to adversity and bouncing back from setbacks	Building resilience	Quest
Cultivating a positive mindset and outlook on the future	Building resilience	Quest
Seeking support and resources to enhance resilience and well-being	Building resilience	Quest
Establishing trusting relationships	Establishing trusting relationships	Equilibrium
Fostering a supportive and inclusive care environment where all voices are valued	Establishing trusting relationships	Equilibrium
Advocating for policies and practices that prioritise patient and family-centred care	Establishing trusting relationships	Equilibrium
Clarifying the roles and responsibilities of each member of the caregiving team	Clarity in roles and responsibilities	Equilibrium
Promoting accountability and transparency in decision-making processes	Clarity in roles and responsibilities	Equilibrium
Creating mechanisms for feedback and continuous quality improvement	Clarity in roles and responsibilities	Equilibrium
Accessing comprehensive support services to address the child’s medical, emotional, and social needs	Holistic support systems	Equilibrium
Engaging with community resources and support networks to enhance overall well-being	Holistic support systems	Equilibrium
Promoting resilience and self-efficacy in caregivers through education and empowerment programs	Holistic support systems	Equilibrium
Advocating for policies and funding to expand access to holistic support services for families of children with complex medical needs	Holistic support systems	Equilibrium
Adapting to changes in the child’s healthcare needs and treatment plans	Flexibility and adaptability	Equilibrium
Advocating for flexible work arrangements and financial supports to accommodate caregivers’ changing circumstances	Flexibility and adaptability	Equilibrium
Respecting the expertise and preferences of all stakeholders	Shared decision-making	Equilibrium
Engaging in open and transparent communication to explore treatment options and make informed choices	Shared decision-making	Equilibrium
Promoting a partnership-based approach to care planning and management	Shared decision-making	Equilibrium
Ensuring consistent access to healthcare services and supports	Continuity and stability	Equilibrium
Facilitating seamless transitions between care settings and providers	Continuity and stability	Equilibrium
Minimising disruptions to the child’s care plan and routines	Continuity and stability	Equilibrium
Promoting stability in the caregiving environment to enhance the child’s well-being	Continuity and stability	Equilibrium
Prioritising caregivers’ own physical and emotional well-being	Self-care and well-being	Equilibrium
Seeking support and resources to cope with the stresses of caregiving	Self-care and well-being	Equilibrium
Promoting a culture of self-care and mutual support within caregiving communities	Self-care and well-being	Equilibrium

Sub-themes and themes identified during thematic analysis and how each was grouped to Frank’s narrative typologies of chaos, quest, and the equilibrium typology uncovered by this study

Most days I am in total fear of what to do and who to contact. I don’t know who to trust or who will help me. The healthcare system seems so complicated, and I feel completely incompetent. (P4, Mother).

One of the major challenges voiced by both parents/guardians and staff, was navigating the complex medical system, particularly when different specialist teams and personnel are involved. Staff told stories of their own difficulty with navigating the system and providing helpful information to families, particularly when they were unsure of what other medical professionals had already told them.

Trying to help these families can be confusing, time-consuming and a bit soul-destroying. I tried to help a family who were having to regularly drive two hours both ways to receive a blood test that I believed could be done more locally. I contacted several different local staff to try and come up with a solution. I don’t know if this should have been my responsibility. But I wanted to help. But in the end, I kept coming up with dead ends and it all got too time-consuming to pursue. (S14, Clinical Nurse Consultant).

The need to shift the focus from provider convenience to patient-centred care was prevalent within staff stories. Staff often used the interview time to critically reflect on the existing processes and systems, and their role in them.

We had a child that came in who had a genetic condition where she had frequent seizures. She was tube fed. Her mum was amazing. She had two other disabled children as well. The challenge for her was that even just getting to the hospital was a mission. So getting the kids into the car, getting them to the hospital, you can’t get a park (…) I just used to feel so sorry for this mum. She’s got so much on her plate. Life is hard. Yet for a little appointment where it might be a blood test or a simple appointment. It’s a 1/2 day mission for her to actually undertake that. How do we make it about the patient and what the patients and their family’s needs are rather than them trying to fit into what is easiest for us? (S2, Clinical Nurse Consultant).

Themes within the chaos narrative typology also highlighted the challenges that families faced when trying to navigate the medical system, particularly when they had more than one chronic or complex condition to manage. Families spoke of experiences being siloed into different specialty areas, making it difficult to keep on top of different appointments and care plans. They voiced disappointment over being expected to manage multiple teams and act as the primary conduit for information across various facilities and departments.

Quite often they will say, because the girls have a vision impairment, “Oh so what does the doctor think about that? How is their vision now?” I’m like well, I don’t know. I feel like it’s important for them to get my perspective, but if they want the proper ‘doctor information’ I feel like that’s something that would be easier communicated doctor to doctor. So having the medical opinion I feel is probably more important than me saying, well, ‘they don’t run into the walls when they walk around’. Like I know that’s good information, but it’s also not. (P7, Mother).

A recurring theme was the profound impact that having a CMC can have on family dynamics, relationships, and social situations. These narratives often revealed the tremendous challenges and adjustments families face in navigating the healthcare system, caregiving responsibilities, and the emotional toll it takes on their interpersonal relationships and broader social connections.

She (sister of CMC) gets quite distressed when her sister is distressed, and she also is quite resentful of missing school or being dragged to appointments these days. I try and avoid that because she misses enough with being a twin sister of a child with a disability. So we try and leave her out of it if we can. But that means we spend less and less time with her. (P7, Mother).

Typical stories illustrating the chaos typology reveal the immense challenge of managing CMC. The constant juggling of appointments, caregiving responsibilities, and inadequate communication with and amongst staff contributed to a feeling of disorder and frustration for everyone involved, including family members and healthcare professionals. Whilst some individuals endured a perpetual state of disorder, others found these experiences to be a catalyst for seeking positive change, as seen in the quest typology.

### Quest typology

*Carmen wanted to spare her 2-year-old son James from undergoing multiple general anaesthetics (GA) for his complex medical needs. Faced with resistance from staff members at her local hospital, Carmen refused to accept the status quo. Determined to find a solution, she wrote a letter addressed to the general manager of the hospital, detailing James’ medical history and the potential benefits of combining procedures under a single GA. Carmen received the gratifying news that her request had been granted. James underwent the necessary procedures without the burden of additional anaesthetics. Carmen even coordinated for James’s dentist to do a check-up while James was under the GA.*

Many stories from staff and families exemplified themes characterised by the quest typology. These themes reflected experiences of pushing past real or perceived barriers in order to reach a positive outcome (see Table 4 for themes identified within the quest typology).

Communication between teams can be average at best. It’s a battle to push past those barriers, because sometimes you might not get an answer with that first email. But you just keep pressing on and you just kind of worm your way in sometimes. And sometimes they probably think you’re a pain in the neck. But if you just keep the patient and their family at the centre of your care you can’t go wrong. It’s just the way it has to be to have the best outcome for that patient and that family member. (S7, Clinical Nurse Consultant).

Many participant stories followed the four stages of the quest typology: (1) the setting out, (2) the struggle, (3) the encounter with the other, and (4) the return. In the example of Carmen and James above, the setting out was the need to avoid multiple GAs for a CMC, the struggle was the reluctance of the hospital to arrange multiple procedures under one GA, and the encounter with the other was the letter written to the general manager. The return was the satisfaction of successfully combining procedures under one GA. Amidst the challenges, there were moments of triumph that arose from the collaborative efforts between healthcare providers and families. A family’s proactive approach, driven by their trust in the healthcare system, became a catalyst for positive change. Seeking help when they feel at their wits’ end, families reached out to healthcare providers, believing that together they could make a difference.

Her central line got blocked and they needed to put a new one in, which meant that it was going to be another surgery (…) I mentioned that she had an MRI scheduled soon and would it be possible to do that at the same time to save her a general anaesthetic. And I was told that it was a good idea, but that it was unlikely that they could do that. I ended up writing a letter to the head of the hospital explaining the situation and how many general anaesthetics she’d had and that this was one time that the (hospital) could really do what’s in the best interest of this child and try and combine those two procedures under the one anaesthetic. And they did it. And not only did they do it that one time, they did it the next time as well. (P1, Mother).

A ubiquitous theme amongst participant stories was the transformative power of effective communication. Parent participants recounted when their concerns were dismissed or undermined and how this taught them that merely smiling and nodding was not effective. They described learning to assert their voices and advocate for loved ones, as they navigated the complexities of healthcare. Empowered by their own experiences, they learnt to approach healthcare interactions with greater confidence and to engage in open and honest dialogue with healthcare providers.

You almost have to know the language. And you have to know what information to give and why it’s important too. (P5, Mother).

Fragmentation in healthcare was seen as a significant issue. Many stories centred on the importance of acknowledging the holistic needs of patients and families beyond their medical needs. Central to these stories was the motivation of the parent/carer to search for answers and solutions – a key component of the quest narrative.

They’ve got a child with a disability and we as the health service have taught them that they have to fight for everything that they need. So we’ve created this ‘difficult parent’ when in fact they’re not actually a difficult parent. They’re just trying to do the best thing for their child. (S15, Clinical Nurse Consultant).

Stories typifying the quest typology illustrate the need to overcome barriers and achieve positive outcomes in managing CMC. Effective communication becomes a transformative force, empowering families to advocate for their loved ones and engage in meaningful dialogue with healthcare providers. Some participants reflected on the sense of fulfilment that their quest provided, while others focused on power balance and role negotiation within the caregiving team, as seen in the equilibrium typology.

### Equilibrium typology

*Tri was diagnosed with a chronic and complex neurological and metabolic condition. Her dad Bianh has been her main carer for over 10 years. In the early days a Doctor said to Bianh, “I’m the doctor, you’re the dad, I know best”. This took his confidence for a long time. Over time, Bianh felt that the different staff members involved in Tri’s care had come to understand Bianh and respected his input. He started to understand the different roles and the differences between the different services that cared for Tri. Bianh learnt that when staying in Ward 1, it was better for everyone if Bianh gave Tri a bath, and he knew which specialists preferred receiving questions from him via email rather than a phone call. Equipped with this knowledge, Bianh now confidently communicates his role and responsibilities to new staff members involved in Tri’s care.*

Through the analysis of interview data, the project team identified that the restitution typology was absent from the narratives of both staff and parents/guardians. In addition, the project team identified one new narrative typology prevalent in the narratives of participants. This typology is ‘equilibrium’. Participant narratives that aligned with this typology typically described medical complexity as a balance of power and a negotiation of roles. They viewed their illness trajectory as a series of negotiations or balancing acts between healthcare stakeholders, before finally reaching some sort of equilibrium. For parents/guardians in particular, this typology encompassed the internal struggle to fulfil the role of an expert in their child’s care, balanced with not wanting the full encumbrance of being the care coordinator for their child (see Table 4 for themes identified within the equilibrium typology).

Within the equilibrium narrative, families grappled with the desire to be knowledgeable advocates for their child’s well-being. They strived to understand the intricacies of their child’s condition and treatment options, becoming experts in their own right. However, they also recognised the significance of not shouldering the entire burden of coordinating care. They sought a balance, where their expertise was respected, whilst maintaining the ability to rely on the guidance and support of professionals who possessed specialised knowledge and skills.

My child had an aspiration event. He became very gravely ill. That was initially managed by the doctors in ED, and I had a very strong sense of what the problem was. I’ll never forget that the doctors really listened to me. It was very late at night and he came in and they, as a team, saved his life. They were really focused on ‘what can this mother tell us that we need to know to help this child’. That to me is really at the heart of the very good care that we get. (P3, Mother).

Staff member participants acknowledged the unique power dynamics that exist within the family member/healthcare professional relationship. They expressed appreciation for the valuable insights and expertise that families brought to the table, but also recognised that this is case-dependent. Equilibrium in their role was about recognising and respecting the capabilities and preferences of the family. The narrative themes lay firmly in comprehending their own roles and responsibilities in a manner that harmoniously aligned with the power dynamics between families and professionals. It was the delicate equipoise between providing professional guidance and expertise whilst acknowledging and incorporating the family’s knowledge and preferences into the care plan.

And when we’re on a stay we do a lot of what the nurses would normally do because you know, it’s what we do every day, so it’s fine. But to then not give us the respect of consulting us a bit more. Like we’re not doctors by any means or anything like that but we do know [child’s name removed] and together we could work really well. (P9, Mother).

One recurring theme was the recognition of the parent/guardian as an important member of the team, as evidenced by their desire to contribute ideas and suggestions. They appreciated when healthcare providers listened to their insights and acted upon them, recognising the crucial role families can play in their child’s well-being. However, limited time and resources could sometimes hinder this collaboration, resulting in missed correspondence and fragmented communication between different healthcare professionals involved in the child’s care. The issue of time constraints was consistently mentioned as a significant factor impacting the quality of care provided. Healthcare professionals were often overloaded with responsibilities, and the limited time available for appointments and consultations restricted their ability to address all aspects of a child’s condition comprehensively.

A lot of my time for my complex patients isn’t recognised as part of my clinical work. It’s hard to actually identify the work involved in making phone calls, sending emails, writing reports, writing scripts, following up medications. All those little bits of time around one patient are not captured by a single appointment in my clinic. I think it’s under-recognized and there is really no robust way of capturing what is required to meet the health needs of these children. (S17, Medical Consultant).

The lack of clarity in roles and responsibilities also emerged as a significant theme. Whilst some parents were proactive and well-informed, describing coordinating their child’s care effectively, others struggled due to the overwhelming demands of caring for a CMC. The absence of a designated coordinator or a clearly defined lead role contributed to confusion and a sense of being caught in the middle for families. This ambiguity led to situations where different clinicians passed the responsibility to others, which caused delays and potential gaps in care. One service manager described that the most complex patients were the ones that missed out most, in that “everyone thinks everyone else is doing it”.

I think some of the challenges at the local level, are passing the buck or believing someone else will sort it out, and it’s not my job. I think it’s unfair for families to be caught in the middle of that, of two clinicians saying well, it’s not my job. And that’s where the families and the patients ultimately suffer in that situation. (S8, Clinical Nurse Consultant).

A prominent theme that emerged from the stories was the evolving dynamics and roles between children, families, and multiple staff members. Within positive experiences shared, a recurring element was the establishment of a mutual understanding between families and staff members regarding their respective functions and capabilities. This understanding fostered effective collaboration and contributed to the overall well-being of CMC and their families.

## Discussion

Our findings reveal the absence of the restitution narrative in discussions concerning CMC, highlighting the unique nature of the clinical and familial caregiving journey. Moreover, we introduce a novel typology termed ‘equilibrium’, shedding light on the delicate balance of roles and power dynamics within this context. Additionally, our study highlights the distinct burdens faced by parents/guardians and staff, emphasising the imperative for tailored support systems. These insights underscore the critical need for integrated, family-centred approaches in caring for CMC, paving the way for improved care coordination and enhanced outcomes.

### Narrative analysis and absence of restitution

Analysing staff and parent/guardian interviews together allowed us to bring these narratives together, in the pursuit of common experience amongst these stakeholders, and to inform development of effective models of care [18, 24, 25]. This is of particular importance where the success and failure of integrated care interventions, including care coordination, have always been context dependent [26–28]. 

Frank’s archetype of restitution has been exemplified in narratives from survivors of cancer, Human Immunodeficiency Virus (HIV), and spinal cord injury [29–32]. However, the restitution narrative is absent from conversations about CMC. The restitution narrative describes coming to a place of healing, wellness, or managed independence, which is not a concept which typically applies to CMC. CMC are highly dependent on parent/guardian support, their conditions oftentimes lifelong and, in many cases, life-limiting. Within high income countries, parent/guardian interactions with the healthcare system can be frequent, long-term and occur across multiple health services.

### Significance of long-term relationships between family and staff

Within our findings, the current limitations to provision of integrated care are starkly exposed, reflecting the findings of previous studies and literature on care provision for the CMC cohort [7–11]. These findings align with previous research [33–35], highlighting the significance of a long-term relationship between family and staff, that is consistently experienced as a source of stability and safety. This relationship serves as a platform for expressing care and addressing broader family needs, where these needs are genuinely heard and respected. The key to a successful long-term relationship lies in fostering an environment of mutual respect and collaboration. These findings also align with the WHO (World Health Organisation) framework on integrated, people-centred health services. This indicates that the future of care requires an ‘equal and reciprocal relationship’ between clinical and non-clinical professionals together with the individuals using care services, their families and communities [36, 37]. 

### Emergence of equilibrium narrative typology

While acknowledging the dynamic nature of Frank’s typologies, particularly the ongoing nature of the restitution narrative [18], our concept of equilibrium stands apart by emphasising the intricate balance and interplay of roles and power dynamics specifically within the context of caring for CMC. While restitution narratives may involve ongoing challenges, and this does overlap somewhat with the equilibrium typology, the later delves deeper into the nuanced interactions and negotiations inherent in the caregiving journey for CMC. Overall, the equilibrium narrative typology highlights the complex and challenging nature of caring for a CMC for both staff and parents/guardians. The theory of illness narrative is closely connected to cultural and structural contexts [18, 24]. Within these contexts, we can observe healthcare staff and parents/guardians in the wider community providing care for individuals whose conditions often fall outside the realm of easily comprehensible illness experiences. As outliers to the common experience, parents/guardians and clinicians uniquely share the proximity and intensity of illness experiences collectively and make meaning within [18], and from, the narrative interdependence [38]. Clinician and parent/guardian narratives equally described the ongoing interactions, and the long-term, emotionally fraught, ethically challenging, and isolating experiences of medical complexity. Despite similarities in experience and perceptions of CMC, clinician and parent/guardian experiences had notable contrasts.

Our findings demonstrate that parents/guardians and staff experienced confusion about the roles and responsibilities of different members of the team. For parents/guardians, understanding this delineation, and therefore their own role within the team was extremely important. This theme has been well documented in the literature [39–44]. The existing body of evidence points to roles as being experienced as transient and malleable within the fragmented service structures which embody healthcare currently. Where roles are more defined, and where care coordination supporting role delineation is resourced and available, outcomes improve [11, 43, 45]. Additionally, these findings show that when roles are clearly defined and communicated, parents/guardians and staff are more confident to act with efficiency and reduced stress and burden on all stakeholders.

### Burden of care experienced by clinicians and families

In these findings, a key difference between staff and parents/guardians is the type of burden they experience. While staff members face the challenge of balancing clinical and non-clinical tasks, an issue commonly noted in literature [43, 45, 46], parents/guardians carry the burden of providing continuous care for their CMC within the complexities of family life, community life, and across an ever-changing environment of healthcare provision [5, 47, 48]. This difference in burden highlights the need for comprehensive support systems that address the emotional, financial, and physical challenges faced by parents/guardians to support better outcomes [49–54]. Consistent with our findings and existing literature, staff members may benefit from streamlined processes and accessible resources to facilitate interpersonal communication and information transfer to fulfil their care coordination responsibilities [28, 45, 55]. 

The findings also highlight the juggling roles of parents/guardians as both a caregiver and a parent. This theme underscores the unique challenge faced by parents/guardians in balancing their roles and responsibilities, which can have implications for their own well-being and the quality of care provided [5, 47–50]. Integrating parents/guardians as part of the care team and providing suitable support and coaching for families, as trialled and recommended in other research, may be effective in addressing this challenge [54–57]. Greater parent/guardian involvement in care for CMC has been found to contribute to more effective care coordination and improved outcomes [58, 59]. Families are key to improved integrated care experiences and these conversations are best placed to be instigated by formal care providers [60]. 

This analysis provides novel insight into the relationship between staff and parents/guardians of CMC. It reflects the shared experience of long-term provision of care in a context where an integrated care approach is essential [11]. Meaning can be drawn from the sharing of staff and parents/guardians narratives and juxtaposing them, at an individual clinician/patient level of care. For instance, when considering how services and systems interact with families to support an integrated care experience and when considering the key aspects of governance, policy, and funding approaches that require a shift to achieve integrated care [11]. 

### Family-centred approaches

The data contributes to a clearer understanding of why family-centred approaches are particularly important for this cohort. This builds on previous work to identify effective family-centred approaches to care for CMC [50, 51, 56]. By forming a comprehensive understanding of the dynamic interplay between CMC, families, and staff and their respective challenges, models of care that better serve the needs of families can be developed. These models must be attentive to staff capabilities and the healthcare environment to ensure practicality. With this pragmatism, models are more likely to be successful and sustainable. Recognising that CMC and their families experience a long-term struggle to balance their role within a large and varied team will help to develop models that are well suited to this cohort [39, 61]. Continuing to enable the parent/guardian voice and adopting a ‘partnering in care’ approach is essential, with new ways to enhance this partnership likely to be discovered and explored [11, 51, 62]. As part of this, the need to address health inequalities that may impede the ability of families to fulfil their role within the care team must also be addressed. Factors such as language barriers or socioeconomic status can hinder effective care coordination for children with medical complexity. Without targeted support, these disparities risk exacerbating existing inequalities in health outcomes [63, 64]. Thus, interventions aimed at mitigating these barriers are essential for ensuring equitable access to quality care for all children and families, regardless of individual circumstances.

### Implications for clinical practice

1. Clinicians should prioritise clear delineation and communication of roles and responsibilities within the caregiving team. This includes ensuring that parents/guardians understand their role within the team, which can lead to increased confidence, efficiency, and reduced stress for all stakeholders.
2. Healthcare organisations and systems need to implement tailored support systems that address the multifaceted challenges faced by parents/guardians of CMC. This includes providing emotional, financial, and physical support to alleviate the burdens associated with continuous caregiving.
3. Recognising the dual roles of parents/guardians as caregivers and parents, healthcare models should actively integrate them as part of the care team. This involves providing suitable support and coaching for families to help them effectively balance their responsibilities and enhance the quality of care provided to CMC.

### Study limitation

The limitation of this study is the absence of CMC perspectives. This restricts the depth of insight into care coordination experiences, possibly overlooking unique themes not expressed by adult participants.

### Study strengths

Our study presents a novel analytic approach within the context of research on complex care needs in children. A key strength of this approach is centralising consumer, carer, and clinicians experience within their own journeys or ‘stories’ of experience. Considering the experiences and stories of clinicians and families through Frank’s narrative typologies has helped to reveal comparisons and contradicts among perceptions and experience. Additionally, a narrative approach to research with CMC allows for the complexity and heterogeneity of CMC experiences to be captured in a linear and graspable way that helps to make sense of these experiences.

## Conclusions

CMC as a group are heterogeneous. They span multiple specialities and multiple services. Our study explores the challenges and dynamics within the caregiving journey for CMC, particularly through the analysis of themes within the equilibrium typology. Our findings emphasise the need for integrated, family-centred approaches in caring for CMC, advocating for tailored support systems and clear delineation of roles within caregiving teams. By understanding and addressing these nuanced needs, healthcare providers and policymakers can work towards more holistic, patient-centred approaches to care, ultimately driving positive change in healthcare systems for CMC and their families.

### Electronic supplementary material

Below is the link to the electronic supplementary material.

Supplementary Material 1

## Data Availability

The datasets analysed during the current study are available from the corresponding author on reasonable request.

## Abbreviations

CMC: children with medical complexity
GA: general anaesthetics
HNELHD: Hunter New England Local Health District
HIV: Human Immunodeficiency Virus
RRMA: Rural, Remote and Metropolitan Area
WHO: World Health Organisation
