# Supplementary material for: Between equilibrium and chaos, with little restitution: a narrative analysis of qualitative interviews with clinicians and parent carers of children with medical complexity
Source: BMC Health Serv Res. 2024 Apr 23;24:504. doi: 10.1186/s12913-024-10973-6 (PMC11040924; doi:10.1186/s12913-024-10973-6)
Supplement: Supplementary file 1 — Supplementary Material 1 [file 12913_2024_10973_MOESM1_ESM.pdf]

Additional file 1: Semi-structured Interview Guides

Guide 1: Health Professional Semi-structured Interview Guide

Topic	Purpose	Questions	Suggested Prompts
**Participant details**	Confirm participant details	• What is your name?<br>• What is your role?<br>• Where do you work?	
**Experience working with medically complex children**	To understand the experience the participant has in working with medically complex children	• Can you describe your experience working with children who have chronic and complex health needs?	• How long have you been in your role?<br>• Have you worked in other roles related to care for medically complex children?
**What is coordinated care**	To understand what coordinated care means to the participant	• What does connected and coordinated care mean to you?	
**During appointments**	To understand the experience of health professionals during appointments	• Can you describe what an average appointment / inpatient ward care / or other interaction with a medically complex child looks like for you?	• How much information about them do you get beforehand?<br>• What kind of things do you discuss with them?
**Health staff communication**	To understand the perception of how well health staff communicate with each other	• In your experience, how do health professionals from different facilities or specialties communicate with each other when treating the same child?	• Is there communication?<br>• How does this happen?
**Positive experience**	To gather positive care coordination experiences	• Can you give an example of when you have experienced or witnessed good care coordination as a professional?	

**Negative experience**	To gather negative care coordination experiences	• Can you give an example of when you have experienced or witnessed poor care coordination as a professional?	
**Barriers to care coordination**	To explore barriers to care coordination	• In your experience, what are the main challenges or barriers to well-coordinated care?	
**Enablers of care coordination**	To explore enablers of care coordination	• In your experience, what do you think would help to make care more coordinated?	
**Anything else**	To give the participant the opportunity to share any other experiences	• Is there anything else you would like to share that we haven't spoken about today?	

Guide 2: Parent/Guardian Semi-structured interview Guide

Topic	Purpose	Questions / Prompts	Suggested Prompts
**Participant details**	Confirm participant details	• What is your name?<br>• Can you please confirm that you are the parent or guardian of a child with chronic and complex health needs?	
**Family context**	Understand some of the home/family context	• Can you tell me a bit about yourself and your family?	• How many children do you have?<br>• Do you work? What do you do?<br>• Do you have a partner? What do they do for work?
**Child needs**	To understand the level of complexity of the child	• Can you please tell me about your child and their health needs?	• How old are they?<br>• What conditions do they have?<br>• What does this mean for your family?
**What is coordinated care**	To understand what coordinated care means to the participant	• What does connected and coordinated care mean to you?	
**Attending appointments**	To understand the experience of families getting to appointments	• Can you tell me about what is involved for your family in getting your child to appointments?	• Are appointments close to where you live?<br>• Do you need to take time away from work to take your child?
**During appointments**	To understand the experience of families during appointments	• Can you describe what happens during an average appointment with a health professional?	• How does the health professional talk to you or your child?

			• What kind of things do you discuss with the health professional?
**Self-management**	To understand the experience of self-management	• What is involved in managing your child's condition at home?	• Do you need special equipment?
**Health staff communication**	To understand the perception of how well health staff communicate about the child	• When your child is seeing multiple specialists or health professionals, how well do you feel they communicate with each other about your child's care?	• Does everyone seem to be on the same page?<br>• Do you have to do a lot of explaining or do they already have information?<br>• Any examples of when this communication went well?
**Negative experiences**	To gather negative care coordination experiences	• Can you please tell me about an experience you have had where care **hasn't** been coordinated well for your child?	
**Positive experiences**	To gather positive care coordination experiences	• Can you please tell me about a time when care **has** been coordinated well for your child?	
**Barriers to care coordination**	To explore barriers to care coordination	• In your experience, what are the main challenges or barriers to well-coordinated care?	
**Enablers of care coordination**	To explore enablers of care coordination	• In your experience, what do you think would help to make care more coordinated?	
**Anything else**	To give the participant the opportunity to share any other experiences	• Is there anything else you would like to share that we haven't spoken about today?	

Guide 3: Child (7-13 Years) Semi-structured Interview Guide

Topic	Purpose	Questions / Prompts	Suggested Additional Prompts
**Participant details**	Confirm participant details	• What is your name?	
**Family context**	Understand some of the home/family context	• Can you please tell me a bit about yourself and your family?	• How old are you?<br>• Do you go to school?<br>• Who lives with you?<br>• What do your parents do for work?
**Child needs**	To understand the level of complexity of the child	• Can you tell me about your health?	• Do you have any health problems?<br>• Do these problems make things tricky for you? How?
**What is coordinated care**	To understand what coordinated care means to the participant	• What does good health care mean to you?	
**Attending appointments**	To understand the challenges and enablers of attending appointments	• Can you tell me about what happens when you need to go to a doctor?	• Do you have to take time away from school?<br>• Do you have to travel very far?
**During appointments**	To understand the experience of families during appointments	• Can you tell me what seeing a doctor is like for you?	• Does the doctor talk to you, your parent or both?<br>• Do they ask you about what you would like for your health?
**Self-management**	To understand the experience of self-management	• What do you do each day to look after your health at home?	• Is there anything special that you need at home to help you?

**Negative experiences**	To gather negative care coordination experiences	• Can you please tell me about a time when you weren't happy with what was happening in hospital?	• How did that make you feel?
**Positive experiences**	To gather positive care coordination experiences	• Can you please tell me about a time when you were happy with what was happening in hospital?	• How did that make you feel?
**Anything else**	To give the participant the opportunity to share any other experiences	• Is there anything else you would like to tell me?	

Guide 4: Young Person (14-17 Years) Semi-structured Interview Guide

Topic	Purpose	Questions / Prompts	Suggested Prompts
**Participant details**	Confirm participant details	• What is your name?	
**Family context**	Understand some of the home/family context	• Can you please tell me a bit about yourself and your family?	• How old are you?<br>• Do you go to school / do you work?<br>• Who lives with you?<br>• What do your parents do for work?
**Child needs**	To understand the level of complexity of the child	• Can you tell me about the health problems that you have?	• What are the problems/conditions you have called?<br>• What does having these conditions mean for you?
**What is coordinated care**	To understand what coordinated care means to the participant	• What does connected and coordinated care mean to you?	
**Attending appointments**	To understand the challenges and enablers of attending appointments	• Can you tell me about what happens when you need to go to a doctor or nurse?	• Do you have to take time away from school/work?<br>• Do you have to travel very far?
**During appointments**	To understand the experience of families during appointments	• Can you talk about what seeing a doctor or nurse is like for you?	• Does the person talk to you, your parent or both?<br>• Do you have a chance to talk about your goals / what you would like for your health?<br>• Is what happens when you start moving to adult care spoken about?

**Self-management**	To understand the experience of self-management	• What do you do each day to look after your health at home?	• Do you need special tools or equipment?
**Negative experiences**	To gather negative care coordination experiences	• Can you please tell me about a time when you weren't happy with your care at an appointment or in hospital?	• How did that make you feel?
**Positive experiences**	To gather positive care coordination experiences	• Can you please tell me about a time when you were happy with your care at an appointment or in hospital?	• How did that make you feel?
**Anything else**	To give the participant the opportunity to share any other experiences	• Is there anything else you would like to share that we haven't spoken about today?	
